# Data on empirically estimated corporate survival rate in Russia

**DOI:** 10.1016/j.dib.2017.12.011

**Published:** 2017-12-16

**Authors:** Evgeny A. Kuzmin

**Affiliations:** Department of Corporate Economics, Ural State University of Economics, Ekaterinburg, Russian Federation

**Keywords:** Corporate survival, Corporate life length, Corporate resistance rate, Life cycle, Business demography

## Abstract

The article presents data on the corporate survival rate in Russia in 1991–2014. The empirical survey was based on a random sample with the average number of non-repeated observations (number of companies) for the survey each year equal to 75,958 (24,236 minimum and 126,953 maximum). The actual limiting mean error ∆*p* was 2.24% with 99% integrity. The survey methodology was based on a cross joining of various formal periods in the corporate life cycles (legal and business), which makes it possible to talk about a conventionally active time life of companies’ existence with a number of assumptions. The empirical survey values were grouped by Russian regions and industries according to the classifier and consolidated into a single database for analysing the corporate life cycle and their survival rate and searching for deviation dependencies in calculated parameters. Preliminary and incomplete figures were available in the paper entitled “Survival Rate and Lifecycle in Terms of Uncertainty: Review of Companies from Russia and Eastern Europe” (Kuzmin and Guseva, 2016) [3]. The further survey led to filtered processed data with clerical errors excluded. These particular values are available in the article. The survey intended to fill a fact-based gap in various fundamental surveys that involved matters of the corporate life cycle in Russia within the insufficient statistical framework. The data are of interest for an analysis of Russian entrepreneurship, assessment of the market development and incorporation risks in the current business environment. A further heuristic potential is achievable through an ability of forecasted changes in business demography and model building based on the representative data set.

**Specifications Table**

**Table:** 

Subject area	*Economics*
More specific subject area	*Business demography, corporate life cycle*
Type of data	*Table*
How data were acquired	*Collection of publicly available registration data on companies and their financial statements*
Data format	*Raw, estimated*
Experimental factors	*A consolidated set of data on companies (grouped by region).*
Experimental features	*The adjusted length of companies’ life in Russia is estimated based on the information on registration changes and financial and operating performance in 1991–2014.*
Data source location	*Ural State University of Economics and Institute of Economics of the Ural Branch of the Russian Academy of Sciences (Ekaterinburg, Russian Federation)*
Data accessibility	*Data available with this article and publicly available online on*https://data.mendeley.com/datasets/7jtxrpkyxx/1*;*
	*Disaggregated data provision is possible on demand by Department of Corporate Economics of the Ural State University of Economics or Division of Regional Industrial Policy and Economic Security of the Institute of Economics of the Ural Branch of the RAS*

**Value of the data**

• The data show the survival rate of Russian companies in 1991–2014 and are a benchmark to measure a degree of the market development, as well as institutional and infrastructure support.
• The data are a descriptor of the corporate business activity and resistance to external and in-house risks.
• The data will be useful for an analysis in various areas of business demography and corporate life cycle.
• It might be possible to use the data to measure a risk of doing business in Russia throughout an observation period with a breakdown by regions and industries. It might be also possible to use them to evaluate favourable conditions for incorporation.
• It is possible to use these data to make a forecast of changes in business demography and for model building.

## 1 Background

The corporate survival rate is an important characteristic of the market development degree. A comparative analysis of the survival rate gives an idea of the institutional and infrastructure support for a certain area. All of these parameters one way or another point out to the uncertainty and risk existing while making transactions, where the high complexity in transactioning generates equally high transaction costs. The environment viscosity faced by companies creates a unique set of dynamic capabilities. The organizational immunity being formed must ultimately be determinative for a length of the corporate life cycle. These particular arguments were a basis for the survey to test a number of hypotheses concerning an importance of the external environment to find a horizon for the corporate survival rate. At the same time, this does not limit the heuristic potential and applications of the figures in business demography, an analysis of life cycles, entrepreneurial activity and viscosity of the economic environment.

The performed empirical experiment for Russia intended to fill the fact-based gap in fundamental survey within the insufficient statistical framework. On the one hand, the Federal State Statistics Service of the Russian Federation [6] gives a narrow set of indicators relating to demography of small and medium-sized businesses and such indicators are not clearly representative for an idea of an average age of companies. On the other hand, similar empirical studies with a small sample and published at different times give non-harmonized values (for example, the average age of companies in Russia was 19.2 years in 2006 as reported by Shirokova [4], in 10 years, in 2016, it was 7.8 years [2]. Others, e.g. Shamray, report that in 2010, the average age of companies was 7.1 years [7]). In case with small set companies included in the survey, sampling generates a high limiting computational error, which does allow extrapolating the identified regularities to the full suite of Russian companies.

**Table 1:** Survival rate of Russian companies in 1991–2014, %.

**Т+**	**1991**	**1992**	**1993**	**1994**	**1995**	**1996**	**1997**	**1998**	**1999**	**2000**	**2001**	**2002**	**2003**	**2004**	**2005**	**2006**	**2007**	**2008**	**2009**	**2010**	**2011**	**2012**	**2013**	**2014**
0									94.2	92.0	92.1	88.5	89.6	91.1	92.1	92.2	91.6	91.9	93.9	92.5	94.6	94.1	89.5	86.0
1								88.6	82.6	79.1	75.7	73.4	77.9	79.5	81.3	82.8	80.6	82.4	82.8	83.8	84.2	77.7	70.4	
2							87.2	77.1	71.4	64.2	63.2	62.9	67.9	70.7	72.2	74.8	72.1	73.5	74.8	74.0	68.4	60.0		
3						88.4	75.7	66.9	59.5	53.9	54.8	54.8	61.2	62.9	64.9	68.6	65.1	67.8	67.8	61.8	54.2			
4					89.5	78.2	65.9	56.0	51.5	47.1	48.1	49.1	55.8	56.9	59.8	63.2	60.8	62.7	58.3	50.5				
5				90.6	79.5	68.9	55.7	48.7	46.0	41.8	43.5	44.6	51.0	52.7	55.5	58.9	56.5	54.6	48.4					
6			92.1	81.8	70.5	58.7	49.0	43.8	41.6	38.0	39.7	40.8	47.6	49.1	52.6	55.2	49.9	46.0						
7		93.2	84.2	73.4	60.3	52.0	44.4	39.9	38.2	34.6	36.5	38.0	44.5	46.8	49.8	49.6	42.5							
8	91.7	86.5	76.0	63.2	53.8	47.4	40.4	36.9	35.2	32.0	33.9	35.4	42.5	44.5	45.0	42.8								
9	84.6	78.7	66.1	56.4	49.0	43.4	37.4	34.3	32.9	29.9	31.8	33.8	40.5	40.3	39.3									
10	77.1	68.7	59.1	51.6	45.0	40.4	34.8	32.1	31.1	28.1	30.4	32.2	37.0	35.4										
11	67.5	61.6	54.2	47.2	42.0	37.8	32.6	30.1	29.4	26.9	28.9	29.4	32.7											
12	61.2	56.3	49.6	43.8	39.3	35.5	30.8	28.5	28.2	25.6	26.4	26.0												
13	57.0	51.2	46.3	41.0	37.0	33.7	29.0	27.4	27.0	23.6	23.3													
14	52.8	47.6	43.5	38.6	35.0	31.9	28.0	26.3	25.1	20.9														
15	50.0	44.5	41.2	36.6	33.0	30.7	26.8	24.5	22.6															
16	47.3	42.0	39.3	34.3	31.8	29.6	24.8	22.1																
17	44.9	40.1	37.2	32.9	30.6	27.5	22.4																	
18	42.9	38.2	35.6	31.6	28.3	24.9																		
19	40.9	36.6	34.3	29.4	25.5																			
20	39.3	35.2	31.9	26.7																				
21	37.7	32.7	28.9																					
22	34.6	29.7																						
23	30.7																							

Empirical statistics																								
K1	0.60	0.58	0.55	0.51	0.48	0.44	0.40	0.37	0.35	0.34	0.35	0.36	0.40	0.43	0.46	0.51	0.52	0.56	0.61	0.65	0.71	0.76	0.82	–
К2	14.3	13.2	12.0	10.8	9.6	8.4	7.2	6.2	5.6	5.1	4.9	4.7	4.8	4.7	4.6	4.6	4.1	3.9	3.6	3.2	2.8	2.3	1.6	–
К3	7.3																							

*Note:* К1 - resistance coefficient of the companies established in year T; К2 - average life length of the companies established in year T; K3 - average life length of companies in 1991–2014.

## 2 Data

The processed data include the aggregated information on the Russian companies, established and operated in 1991–2015, the survey also optionally covered the 1987–1990 period. The figures were grouped into three categories: 1) general data without their features considered; 2) data by type of economic activity (according to OKVED v.2 - Russian Standard Industrial Classification of Economic Activities both for ‘old’ and ‘new’ companies with the applied pattern for a transition between OKVED versions); 3) data in the context of companies’ distribution by regions of the country. Main computational parameters within the empirical survey include the information on a number of newly established companies; a natural loss of newly established companies (cumulatively and annually); a specific corporate survival rate; an adjusted and unadjusted life length. There is a further estimation of the annual companies’ resistance (with the value as an indicator of the specific actual life length against the longest possible life length of a company for a given period), as well as the average life length of companies by year of their establishment and in whole throughout the sample for the whole time of observations. Brief primary figures [3] were provisional and did not cover the regional distribution by federal district (Table 2, Table 3, Table 4, Table 5, Table 6, Table 7, Table 8, Table 9), actor, and activity (data are available on demand).

**Fig. 1:**
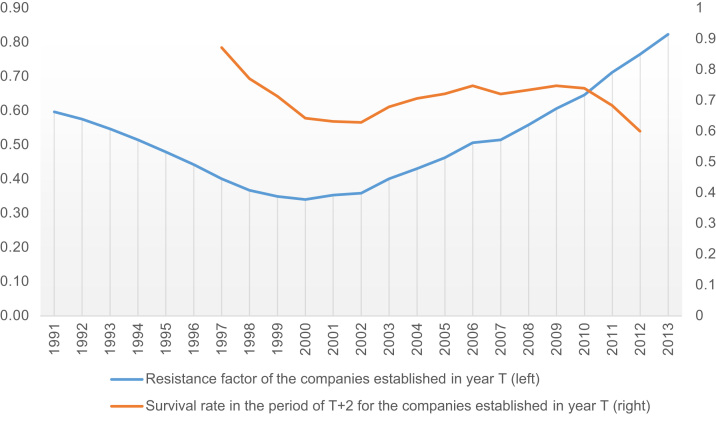
Survival and resistance rate of Russian companies in 1991–2013.

**Table 2:** Survival rate of Russian companies in the Far Eastern Federal District in 1991–2014, %.

**Т+**	**1991**	**1992**	**1993**	**1994**	**1995**	**1996**	**1997**	**1998**	**1999**	**2000**	**2001**	**2002**	**2003**	**2004**	**2005**	**2006**	**2007**	**2008**	**2009**	**2010**	**2011**	**2012**	**2013**	**2014**
0									94.3	92.4	91.9	87.1	87.5	88.9	91.2	93.8	92.7	92.7	94.9	94.1	95.9	95.7	90.8	87.5
1								89.0	82.2	78.6	73.5	69.6	73.7	76.4	81.5	85.2	82.4	85.2	86.2	86.7	88.3	80.5	71.8	
2							87.6	78.4	70.1	61.9	59.9	58.9	64.6	68.5	73.9	78.5	75.6	78.3	80.5	79.8	73.7	63.2		
3						88.3	76.3	68.1	56.4	51.6	50.9	51.6	58.4	61.8	67.8	73.8	70.5	73.8	74.6	67.6	59.7			
4					87.2	77.6	67.3	54.7	48.2	44.3	44.8	46.8	53.3	57.0	64.2	69.5	66.6	69.6	65.1	56.3				
5				90.3	77.6	67.9	56.2	46.7	42.3	39.2	40.4	42.0	49.0	53.4	59.8	65.6	63.0	61.0	54.2					
6			91.6	82.0	68.5	56.1	48.6	42.3	38.5	36.3	37.0	38.7	46.5	50.5	57.7	62.4	55.5	51.6						
7		90.9	83.3	73.7	56.3	48.6	42.4	38.6	35.7	33.3	34.6	36.5	43.8	48.7	55.4	56.5	47.0							
8	91.3	83.3	74.1	62.8	49.7	43.6	38.7	35.9	32.9	30.8	32.4	34.1	42.1	46.8	50.3	49.1								
9	84.7	74.8	63.5	56.0	44.5	39.5	35.8	33.4	30.7	29.1	30.4	32.7	40.6	42.5	44.2									
10	75.9	64.2	55.9	50.5	41.1	37.0	33.2	31.3	29.1	27.5	29.3	31.5	37.1	37.3										
11	64.3	57.1	51.3	46.4	38.8	34.7	30.9	29.5	27.8	26.5	28.4	28.6	32.6											
12	58.0	52.2	47.3	43.1	36.5	32.8	29.5	28.1	26.7	25.4	25.7	25.3												
13	54.0	47.3	44.2	39.8	35.1	31.1	27.7	27.4	25.9	23.2	22.3													
14	49.0	44.1	40.9	37.2	33.6	29.3	26.7	26.7	24.2	20.5														
15	46.0	41.5	38.9	35.7	31.9	28.5	25.7	24.8	21.9															
16	43.5	38.8	37.5	34.0	30.7	27.7	23.8	22.4																
17	41.2	37.7	35.9	32.6	29.3	25.5	21.4																	
18	39.3	35.9	34.6	31.7	27.4	22.9																		
19	37.0	34.8	33.6	29.7	24.4																			
20	36.2	33.6	31.3	27.2																				
21	35.0	31.6	28.8																					
22	33.1	29.1																						
23	29.5																							

Empirical statistics																								
K1	0.58	0.55	0.53	0.51	0.46	0.43	0.40	0.36	0.33	0.33	0.34	0.34	0.38	0.41	0.47	0.53	0.54	0.59	0.64	0.67	0.74	0.78	0.84	–
К2	13.9	12.7	11.7	10.6	9.3	8.2	7.2	6.1	5.3	4.9	4.7	4.4	4.5	4.5	4.7	4.8	4.3	4.1	3.8	3.4	3.0	2.4	1.7	–
К3	7.1																							

*Note:* К1 - resistance coefficient of the companies established in year T; К2 - average life length of the companies established in year T; K3 - average life length of companies in 1991–2014.

**Table 3:** Survival rates of Russian companies in the Volga Federal District in 1991–2014, %.

**Т+**	**1991**	**1992**	**1993**	**1994**	**1995**	**1996**	**1997**	**1998**	**1999**	**2000**	**2001**	**2002**	**2003**	**2004**	**2005**	**2006**	**2007**	**2008**	**2009**	**2010**	**2011**	**2012**	**2013**	**2014**
0									95.1	91.3	91.6	88.0	89.2	90.3	91.2	92.0	91.1	90.8	93.4	91.4	93.5	93.1	88.9	84.5
1								90.0	84.4	78.0	74.9	72.3	76.8	78.0	80.1	82.4	79.6	80.7	80.8	80.9	81.8	75.9	69.1	
2							87.9	78.3	73.4	62.8	62.2	61.6	66.2	68.8	71.2	73.8	70.0	71.2	71.3	69.9	65.7	58.1		
3						88.4	75.3	67.0	61.3	52.1	53.8	53.4	59.1	61.3	64.0	67.1	62.5	64.5	63.7	58.4	51.7			
4					89.9	76.5	65.0	55.0	52.4	44.8	46.8	47.4	53.5	55.1	58.4	61.3	57.8	58.7	54.7	47.6				
5				90.3	79.2	67.2	54.5	47.0	46.4	39.6	42.0	42.6	49.1	50.6	53.6	56.6	53.2	51.0	45.2					
6			91.8	80.5	70.3	56.4	47.3	41.8	41.3	35.7	38.3	38.8	45.4	46.6	50.3	52.5	46.9	42.8						
7		94.0	83.0	71.9	60.7	49.6	42.3	37.7	37.2	32.6	34.9	36.0	42.2	44.1	47.3	46.9	40.0							
8	91.6	87.2	74.5	61.4	53.9	44.6	37.7	34.6	34.3	30.1	32.1	33.2	40.0	41.7	42.5	40.5								
9	84.2	78.8	64.0	54.0	48.6	40.3	34.5	32.0	32.0	28.1	30.0	31.4	37.9	37.6	37.0									
10	76.5	69.1	56.9	48.7	43.4	37.2	31.9	29.8	29.9	26.1	28.3	29.7	34.6	33.1										
11	66.5	61.6	51.4	43.6	40.0	34.5	29.7	27.2	28.1	24.8	26.8	27.1	30.7											
12	59.2	55.3	46.3	39.6	37.1	32.0	28.0	25.6	26.8	23.6	24.5	24.1												
13	54.3	48.8	42.3	37.0	34.6	30.1	26.1	24.6	25.6	21.5	21.8													
14	49.2	44.5	39.5	34.6	32.4	28.1	24.9	23.5	23.7	19.0														
15	45.5	41.0	37.1	32.4	30.0	26.9	23.8	21.8	21.5															
16	41.9	38.6	35.1	30.1	28.7	25.8	21.8	19.7																
17	39.4	36.4	33.0	28.6	27.3	23.7	19.7																	
18	37.0	34.4	31.4	27.2	24.9	21.4																		
19	34.8	33.0	30.0	25.2	22.3																			
20	32.8	31.3	27.8	22.8																				
21	31.1	29.0	25.1																					
22	28.7	26.1																						
23	25.7																							

Empirical statistics																								
K1	0.59	0.58	0.54	0.51	0.49	0.44	0.40	0.37	0.36	0.33	0.35	0.35	0.39	0.42	0.46	0.50	0.50	0.54	0.59	0.62	0.69	0.75	0.82	–
K2	14.2	13.3	11.9	10.7	9.7	8.3	7.2	6.2	5.7	5.0	4.9	4.6	4.7	4.6	4.6	4.5	4.0	3.8	3.5	3.1	2.8	2.3	1.6	–
К3	7.0																							

*Note:* К1 - resistance coefficient of the companies established in year T; К2 - average life length of the companies established in year T; K3 - average life length of companies in 1991–2014.

**Table 4:** Survival rates of Russian companies in the North-West Federal District in 1991–2014, %.

**Т+**	**1991**	**1992**	**1993**	**1994**	**1995**	**1996**	**1997**	**1998**	**1999**	**2000**	**2001**	**2002**	**2003**	**2004**	**2005**	**2006**	**2007**	**2008**	**2009**	**2010**	**2011**	**2012**	**2013**	**2014**
0									93.5	92.7	92.8	89.1	90.5	92.2	93.4	93.2	92.6	93.5	94.9	93.7	96.1	95.4	90.2	87.2
1								85.9	83.2	80.7	77.2	74.6	80.0	82.1	84.3	84.5	82.9	85.7	86.0	87.0	87.5	80.5	72.5	
2							86.1	75.1	72.8	66.2	65.5	65.3	71.4	74.0	76.7	77.4	75.6	78.6	80.1	78.3	73.1	63.8		
3						86.6	74.8	66.6	61.5	56.2	57.9	58.8	65.1	67.2	70.4	71.8	69.6	74.2	73.6	66.0	59.1			
4					88.4	77.8	65.7	56.3	54.1	50.3	52.0	53.6	60.1	61.3	66.0	67.2	66.2	69.6	62.7	54.1				
5				89.4	79.1	68.8	56.5	49.5	49.1	45.1	47.7	49.8	55.6	57.6	62.4	63.3	62.4	61.0	52.9					
6			90.4	81.4	71.1	58.9	50.5	44.9	45.0	41.1	44.0	46.4	52.0	54.7	59.7	60.0	55.4	51.9						
7		91.3	82.7	73.4	61.4	53.6	46.8	41.7	41.7	37.8	40.7	43.6	49.0	52.5	57.1	54.1	47.3							
8	90.0	83.9	75.4	64.5	55.1	49.5	43.1	38.6	38.8	35.2	38.2	41.3	47.1	50.1	52.2	47.4								
9	82.0	76.1	66.8	57.9	50.9	45.8	40.1	35.9	36.4	33.0	36.1	39.9	45.2	45.2	45.5									
10	74.0	67.2	61.0	54.1	47.3	42.8	37.1	33.5	34.9	31.2	34.7	37.9	41.1	39.9										
11	65.5	61.6	57.2	50.5	44.6	40.2	34.7	31.9	32.9	29.9	33.1	34.6	36.0											
12	60.2	57.7	54.4	47.5	42.0	37.6	33.0	30.0	31.6	28.5	30.4	30.9												
13	56.3	54.3	51.9	44.9	39.7	35.9	30.9	29.0	30.3	26.4	26.9													
14	53.1	51.4	49.5	43.0	37.8	33.9	29.7	27.8	28.2	23.7														
15	51.1	49.0	47.5	40.9	35.2	32.6	28.6	25.9	25.7															
16	48.9	46.8	45.7	37.1	34.0	31.5	26.6	23.4																
17	46.5	44.7	42.6	36.1	32.7	29.1	24.2																	
18	44.9	43.2	41.4	35.0	30.5	26.3																		
19	43.3	41.9	40.3	32.9	27.7																			
20	42.1	40.6	37.4	29.8																				
21	41.0	37.6	33.8																					
22	37.8	34.1																						
23	33.5																							

Empirical statistics																								
K1	0.58	0.57	0.55	0.52	0.48	0.45	0.40	0.37	0.36	0.35	0.37	0.37	0.42	0.45	0.49	0.52	0.54	0.59	0.64	0.67	0.74	0.78	0.82	–
K2	14.0	13.1	12.1	10.9	9.6	8.5	7.2	6.2	5.7	5.2	5.1	4.8	5.1	5.0	4.9	4.7	4.3	4.1	3.8	3.4	2.9	2.3	1.6	–
К3	7.5																							

*Note:* К1 - resistance coefficient of the companies established in year T; К2 - average life length of the companies established in year T; K3 - average life length of companies in 1991–2014.

**Table 5:** Survival rate of Russian companies in the North Caucasus Federal District in 1991–2014, %.

**Т+**	**1991**	**1992**	**1993**	**1994**	**1995**	**1996**	**1997**	**1998**	**1999**	**2000**	**2001**	**2002**	**2003**	**2004**	**2005**	**2006**	**2007**	**2008**	**2009**	**2010**	**2011**	**2012**	**2013**	**2014**
0									95.9	95.3	92.3	90.5	91.5	92.9	92.2	94.1	94.0	93.4	96.5	96.5	96.7	95.6	88.4	82.9
1								91.3	87.5	85.1	76.6	77.0	81.2	84.3	82.4	88.3	86.0	87.3	90.7	91.7	90.4	80.2	68.8	
2							89.4	82.3	78.1	71.8	65.0	67.9	73.2	76.0	76.1	82.4	81.1	82.1	85.5	85.2	75.7	62.8		
3						91.1	81.3	73.3	65.9	62.0	58.2	60.8	66.6	69.6	70.3	78.5	75.9	79.0	80.6	72.4	58.3			
4					91.9	82.7	71.4	62.8	58.0	54.5	53.2	55.7	62.4	65.0	66.3	72.5	72.6	75.6	69.1	58.5				
5				91.9	83.6	73.7	59.7	55.5	53.0	49.3	49.0	52.4	58.3	61.3	62.7	68.7	68.7	65.2	55.4					
6			93.3	85.4	73.6	63.4	52.3	50.7	48.9	45.2	45.5	49.0	55.6	57.4	60.4	64.8	60.4	53.8						
7		93.6	86.3	77.4	62.7	56.4	48.7	46.0	45.4	41.7	43.4	46.0	52.9	55.7	58.0	56.6	50.2							
8	92.1	87.5	78.8	68.0	55.7	51.2	44.1	43.0	42.5	39.4	41.4	43.7	51.5	53.9	51.3	45.7								
9	85.8	78.9	68.7	60.9	51.5	46.0	40.3	40.0	40.5	37.6	39.0	41.8	49.5	48.4	43.0									
10	77.9	67.5	61.7	56.6	46.5	42.0	37.8	37.9	39.0	35.9	37.9	39.5	45.4	41.1										
11	66.3	60.7	56.7	52.7	42.8	39.2	35.5	36.1	36.8	34.3	36.5	35.6	38.2											
12	59.0	55.5	51.3	48.5	40.0	37.1	33.6	34.4	35.7	32.9	33.1	29.7												
13	53.8	49.5	47.0	45.7	37.5	35.7	31.8	32.8	34.6	30.1	28.3													
14	48.1	45.7	44.1	42.7	35.5	34.2	30.5	31.6	31.9	25.6														
15	44.8	42.2	41.7	40.9	33.8	32.8	29.5	28.5	27.1															
16	42.4	38.8	40.2	39.4	32.6	32.2	27.1	25.5																
17	40.5	36.9	38.4	37.4	30.8	29.8	23.7																	
18	39.1	34.8	36.4	36.0	28.4	26.2																		
19	37.0	33.4	35.3	32.7	24.6																			
20	35.8	32.3	32.5	28.5																				
21	34.6	29.7	28.3																					
22	31.5	26.1																						
23	27.2																							

Empirical statistics																								
K1	0.59	0.58	0.56	0.54	0.50	0.46	0.43	0.40	0.39	0.39	0.37	0.41	0.44	0.48	0.51	0.60	0.60	0.64	0.71	0.74	0.78	0.78	0.81	–
K2	14.2	13.4	12.4	11.4	10.0	8.8	7.7	6.9	6.3	5.8	5.2	5.3	5.3	5.3	5.1	5.4	4.8	4.5	4.3	3.7	3.1	2.3	1.6	–
К3	8.9																							

*Note:* К1 - resistance coefficient of the companies established in year T; К2 - average life length of the companies established in year T; K3 - average life length of companies in 1991–2014.

**Table 6:** Survival rate of Russian companies in the Siberian Federal District in 1991 - 2014, %.

**Т+**	**1991**	**1992**	**1993**	**1994**	**1995**	**1996**	**1997**	**1998**	**1999**	**2000**	**2001**	**2002**	**2003**	**2004**	**2005**	**2006**	**2007**	**2008**	**2009**	**2010**	**2011**	**2012**	**2013**	**2014**
0									91.1	90.2	89.6	84.8	85.9	88.4	90.2	91.4	89.2	89.6	92.8	90.7	93.9	93.7	89.6	85.2
1								84.6	76.1	74.8	69.4	66.8	71.6	74.2	78.7	80.2	76.2	78.5	79.5	80.6	83.2	77.2	69.7	
2							83.8	70.5	63.1	57.6	55.7	55.6	60.4	64.4	68.0	71.1	66.6	68.3	70.5	70.8	66.4	58.7		
3						85.5	70.3	59.4	50.4	47.0	47.1	47.9	54.0	56.1	60.3	64.2	59.2	62.2	63.4	58.5	51.9			
4					87.4	74.3	59.2	48.2	42.8	40.2	41.0	42.6	48.9	50.2	54.8	58.3	54.8	57.1	53.7	47.2				
5				89.6	76.3	64.4	48.3	41.1	38.3	34.7	36.5	38.4	44.3	46.0	50.3	53.5	50.5	49.2	44.2					
6			91.5	79.8	66.3	53.0	41.4	36.6	34.4	31.4	33.0	34.9	41.1	42.6	47.4	49.7	44.2	41.2						
7		92.0	83.6	70.3	54.8	45.8	37.2	32.7	31.5	28.2	30.3	32.1	38.2	40.3	44.5	44.3	37.4							
8	91.1	84.4	74.5	57.8	48.0	41.3	33.5	30.1	29.2	25.9	27.9	29.7	36.0	38.2	40.0	37.7								
9	83.4	75.9	62.9	50.2	43.3	37.6	31.2	27.9	27.2	24.1	26.1	28.4	34.1	34.7	34.7									
10	75.7	63.3	54.5	45.5	39.5	35.1	29.0	26.2	25.6	22.5	24.7	27.0	30.8	30.6										
11	65.3	55.7	48.9	41.0	36.7	32.9	27.3	24.7	24.1	21.5	23.6	24.7	27.4											
12	58.5	50.4	44.2	38.0	34.3	30.9	25.7	23.4	23.0	20.4	21.3	22.1												
13	54.3	46.0	41.2	35.6	32.3	29.2	24.1	22.4	22.1	18.6	19.1													
14	50.6	42.9	38.7	33.3	30.2	27.5	23.2	21.5	20.4	16.4														
15	48.6	40.2	36.3	31.3	28.7	26.5	22.1	19.9	18.0															
16	46.1	37.6	34.5	29.0	27.6	25.4	20.3	18.1																
17	44.0	35.7	32.3	27.7	26.7	23.8	18.6																	
18	42.0	34.0	30.7	26.4	24.7	21.9																		
19	40.2	32.6	29.4	24.5	21.9																			
20	38.9	31.3	27.2	22.1																				
21	37.2	29.1	24.7																					
22	33.0	26.5																						
23	28.6																							

Empirical statistics																								
K1	0.60	0.56	0.54	0.50	0.46	0.42	0.37	0.33	0.31	0.31	0.32	0.32	0.36	0.39	0.44	0.49	0.48	0.53	0.58	0.62	0.71	0.77	0.83	–
K2	14.3	12.8	11.8	10.5	9.2	7.9	6.7	5.6	5.0	4.7	4.4	4.2	4.4	4.3	4.4	4.4	3.9	3.7	3.5	3.1	2.8	2.3	1.7	–
К3	6.7																							

*Note:* К1 - resistance coefficient of the companies established in year T; К2 - average life length of the companies established in year T; K3 - average life length of companies in 1991–2014.

**Table 7:** Survival rate of Russian companies in the Urals Federal District in 1991–2014, %.

**Т+**	**1991**	**1992**	**1993**	**1994**	**1995**	**1996**	**1997**	**1998**	**1999**	**2000**	**2001**	**2002**	**2003**	**2004**	**2005**	**2006**	**2007**	**2008**	**2009**	**2010**	**2011**	**2012**	**2013**	**2014**
0									94.9	89.3	92.7	88.5	89.7	90.5	90.9	91.9	91.5	92.6	94.7	92.7	95.2	93.7	88.5	85.1
1								87.5	79.8	76.8	75.9	73.8	76.8	77.3	79.6	81.8	80.9	82.7	84.4	86.0	83.7	77.5	68.6	
2							86.5	73.5	68.8	62.7	63.2	63.1	66.6	66.9	70.7	73.5	72.0	72.6	76.8	75.6	67.6	59.4		
3						88.1	72.7	63.4	57.0	53.4	55.0	54.3	59.2	59.6	63.6	66.8	63.5	66.8	68.8	62.0	53.4			
4					88.7	75.5	63.0	53.7	50.0	46.8	47.8	48.0	53.5	53.9	58.3	61.3	59.3	61.3	59.3	50.0				
5				89.8	76.9	66.0	53.8	47.3	44.3	41.7	42.9	43.2	48.7	49.3	54.3	57.1	54.9	53.5	49.4					
6			91.0	79.8	67.5	56.6	48.2	42.2	40.4	37.4	38.7	39.3	45.4	46.2	51.1	53.2	48.7	45.1						
7		91.8	81.2	70.5	57.1	50.5	43.8	38.4	37.2	34.2	35.6	36.5	42.4	44.0	48.0	47.9	41.5							
8	89.8	83.2	72.6	60.6	51.4	46.6	40.1	35.6	34.3	31.4	33.2	34.3	40.5	41.9	43.8	41.2								
9	80.4	75.0	63.6	54.3	47.1	42.8	37.1	33.0	32.0	29.7	31.3	32.8	38.7	38.6	38.5									
10	73.1	65.1	57.4	50.2	43.9	39.8	34.9	30.9	30.6	27.6	30.0	31.2	35.6	33.4										
11	63.7	59.2	53.2	46.3	41.1	37.5	32.9	29.4	29.1	26.4	28.4	28.6	31.3											
12	58.0	54.4	49.0	43.2	38.0	35.2	31.0	27.8	28.1	25.3	26.1	25.3												
13	54.5	50.2	45.9	39.9	36.0	33.5	29.3	26.4	27.0	23.5	23.1													
14	50.4	46.7	43.3	37.9	34.4	32.0	28.2	25.3	25.2	20.6														
15	48.0	43.8	41.2	35.7	32.7	30.6	27.2	23.7	22.7															
16	45.5	41.3	39.5	33.5	31.2	29.7	25.5	21.1																
17	43.3	39.5	37.5	32.3	30.2	27.5	23.1																	
18	41.0	37.5	36.2	31.0	28.2	25.0																		
19	39.3	36.0	34.9	29.0	26.0																			
20	37.8	34.5	32.7	25.8																				
21	36.7	32.3	29.6																					
22	34.1	29.4																						
23	30.8																							

Empirical statistics																								
K1	0.58	0.56	0.53	0.51	0.46	0.43	0.39	0.36	0.34	0.33	0.35	0.35	0.39	0.41	0.45	0.50	0.51	0.56	0.62	0.66	0.71	0.76	0.82	–
K2	13.8	12.9	11.8	10.7	9.2	8.2	7.0	6.1	5.4	5.0	4.9	4.6	4.7	4.5	4.5	4.5	4.1	3.9	3.7	3.3	2.9	2.3	1.6	–
К3	7.5																							

*Note:* К1 - resistance coefficient of the companies established in year T; К2 - average life length of the companies established in year T; K3 - average life length of companies in 1991–2014.

**Table 8:** Survival rate of Russian companies in the Central Federal District in 1991–2014, %.

**Т+**	**1991**	**1992**	**1993**	**1994**	**1995**	**1996**	**1997**	**1998**	**1999**	**2000**	**2001**	**2002**	**2003**	**2004**	**2005**	**2006**	**2007**	**2008**	**2009**	**2010**	**2011**	**2012**	**2013**	**2014**
0									95.1	94.0	93.6	90.9	92.2	93.4	94.5	91.6	92.8	93.2	93.6	93.3	94.6	94.2	89.8	85.4
1								90.1	85.7	82.4	80.0	78.5	83.2	84.3	82.7	82.8	82.3	83.7	83.1	84.6	84.2	77.4	70.9	
2							88.0	81.0	74.7	68.7	68.0	67.9	73.6	76.6	73.1	74.9	74.0	74.7	75.1	74.5	68.1	59.7		
3						89.8	78.8	71.1	63.3	58.2	59.1	58.9	67.0	67.7	65.2	68.7	66.9	68.9	68.3	62.4	54.0			
4					90.3	81.5	69.1	61.1	55.3	51.2	51.9	52.7	61.0	61.1	59.9	63.7	62.4	63.8	59.2	51.4				
5				91.4	81.6	71.8	58.9	53.5	49.5	46.0	47.0	47.8	55.4	56.6	55.4	59.8	58.2	55.9	49.7					
6			93.0	83.7	72.7	62.2	52.4	48.6	45.1	41.6	42.6	43.6	51.8	52.7	52.7	56.3	51.5	47.6						
7		94.2	86.4	75.5	62.6	55.5	47.8	44.7	41.3	37.6	38.9	40.6	48.5	50.3	50.1	50.8	44.3							
8	92.9	88.6	78.5	65.4	56.2	50.8	43.7	41.4	37.9	34.6	36.1	37.8	46.2	47.8	45.1	44.3								
9	87.0	81.5	68.8	58.8	51.1	46.9	40.4	38.4	35.1	32.2	33.8	36.0	44.3	43.2	39.5									
10	80.3	71.8	62.3	53.8	47.2	43.5	37.4	36.0	33.1	30.2	32.2	34.2	40.4	37.9										
11	71.3	64.8	57.3	49.5	44.1	40.7	34.9	34.0	31.1	28.9	30.5	31.1	35.8											
12	65.7	59.4	52.8	46.4	41.3	38.4	32.8	32.2	29.9	27.4	27.9	27.5												
13	62.0	54.1	49.5	43.4	38.7	36.2	30.9	30.9	28.6	25.2	24.6													
14	58.4	50.4	46.7	41.1	36.6	34.4	29.9	29.7	26.6	22.4														
15	55.4	47.3	44.2	38.8	34.6	32.9	28.7	27.6	24.0															
16	53.0	44.9	42.3	37.1	33.3	31.7	26.6	24.6																
17	50.5	43.0	40.2	35.5	32.1	28.9	23.7																	
18	48.5	41.0	38.7	34.3	29.8	26.0																		
19	46.5	39.5	37.3	32.0	26.8																			
20	44.7	38.1	34.8	29.3																				
21	43.0	35.4	31.8																					
22	39.4	32.3																						
23	34.9																							

Empirical statistics																								
K1	0.62	0.59	0.56	0.52	0.49	0.46	0.42	0.39	0.37	0.36	0.38	0.38	0.43	0.46	0.47	0.50	0.52	0.56	0.60	0.65	0.71	0.77	0.82	–
K2	14.8	13.5	12.2	10.9	9.8	8.8	7.5	6.7	5.9	5.4	5.3	5.0	5.2	5.1	4.7	4.5	4.2	3.9	3.6	3.2	2.8	2.3	1.6	–
К3	7.4																							

*Note:* К1 - resistance coefficient of the companies established in year T; К2 - average life length of the companies established in year T; K3 - average life length of companies in 1991–2014.

**Table 9:** Survival rate of Russian companies in the Southern Federal District in 1991 - 2014, %.

**Т+**	**1991**	**1992**	**1993**	**1994**	**1995**	**1996**	**1997**	**1998**	**1999**	**2000**	**2001**	**2002**	**2003**	**2004**	**2005**	**2006**	**2007**	**2008**	**2009**	**2010**	**2011**	**2012**	**2013**	**2014**
0									94.6	92.9	93.4	90.2	90.4	92.1	92.1	94.0	91.8	92.0	94.9	93.2	95.1	94.0	89.9	90.6
1								91.6	83.4	81.0	80.0	76.4	80.1	80.5	83.2	84.6	81.5	83.5	84.4	85.4	84.7	77.1	71.9	
2							90.6	80.5	72.8	68.5	68.8	66.5	70.3	72.1	74.0	77.3	74.2	74.5	76.6	76.3	68.9	61.0		
3						91.5	80.3	70.9	62.3	59.1	60.2	58.6	64.0	64.7	67.3	71.8	68.1	69.5	69.9	64.0	55.7			
4					91.8	82.7	71.5	61.7	54.5	52.6	53.1	53.5	58.5	59.1	62.9	66.3	63.5	64.6	60.5	52.5				
5				92.0	83.1	74.8	63.2	54.8	49.4	47.4	48.8	48.9	53.9	55.4	59.2	61.7	59.2	55.6	49.5					
6			93.2	84.0	74.6	66.7	56.3	49.6	44.8	43.9	45.2	44.7	50.7	52.4	56.2	58.2	52.2	47.2						
7		94.0	85.9	77.2	66.0	60.3	51.8	45.3	41.8	40.2	42.1	41.6	47.8	50.0	53.3	52.1	44.7							
8	93.5	88.2	78.6	69.2	59.8	56.1	48.5	42.2	38.5	37.9	39.8	39.4	45.8	47.5	48.3	44.9								
9	86.3	81.6	70.2	63.4	56.1	51.7	45.6	39.7	36.3	36.0	37.9	37.8	43.8	42.8	42.7									
10	79.1	73.1	63.6	58.9	52.4	48.5	43.0	37.4	35.0	34.5	36.4	36.2	40.4	38.0										
11	70.5	65.4	59.1	54.3	49.6	46.0	40.7	35.3	33.5	33.2	34.7	33.4	36.2											
12	64.2	60.8	54.1	50.9	47.1	43.6	39.1	33.7	32.1	32.0	32.1	29.9												
13	60.2	56.1	50.6	47.9	44.6	42.0	37.8	32.6	30.9	30.1	28.0													
14	55.9	52.6	47.6	45.0	42.7	40.6	36.8	31.4	29.0	27.0														
15	53.1	49.7	44.9	43.2	41.1	39.6	35.4	29.4	26.2															
16	50.1	46.8	42.9	41.1	39.7	38.5	33.3	27.0																
17	47.6	44.8	40.8	39.4	38.7	36.8	30.0																	
18	45.4	42.9	38.9	37.8	36.3	34.2																		
19	43.2	40.7	37.4	35.3	33.3																			
20	41.4	38.9	34.9	32.5																				
21	39.3	36.0	31.7																					
22	36.7	33.0																						
23	33.1																							

Empirical statistics																								
K1	0.61	0.59	0.56	0.53	0.49	0.46	0.42	0.38	0.36	0.35	0.38	0.37	0.41	0.44	0.47	0.53	0.53	0.57	0.62	0.66	0.71	0.75	0.82	–
K2	14.5	13.6	12.4	11.2	9.9	8.7	7.6	6.5	5.7	5.3	5.3	4.8	4.9	4.8	4.7	4.8	4.2	4.0	3.7	3.3	2.8	2.3	1.6	–
К3	8,1																							

*Note:* К1 - resistance coefficient of the companies established in year T; К2 - average life length of the companies established in year T; K3 - average life length of companies in 1991–2014.

The paper presents the data on the overall survival rate of Russian companies in 1991–2014 (Table 1) upon filtering by the adjusted length of the life cycle (2015 is recognised as a final year for the verification). Figure one shows the corporate survival rate in T+2 period (T reporting year when the company was incorporated, +2 years upon the incorporation) for a comparative analysis based on Eurostat statistics (Business Demography [1]) or other sources. The literature review shows that this very period (|2|) is the most popular, but in different models they use either T-2 format or T+2 format that complicate the comparison. The empirical data, owing to a wide coverage, have made it possible to obtain calculated values up to T+23 in the main sample and up to T+27 including the optional sample.

The paper does not include the author's subjective opinion and analytical conclusions regarding the presented values. This allows other researchers to interpret the data independently.

## 3 Experimental design, materials and methods

### 3.1 Study area

The empirical study was focused on gathering the primary data on companies regardless of their revenue and staff numbers and based on the random sampling principle with the achieved target coverage level of up to 10,000 organizations per region per survey year with repetitions. If there were less than 10,000 companies, then all the available data were accepted. For a general totality and overall sample, only those companies were considered that were present on the StatRegister as of a year of their incorporation (based on data provided by the Federal State Statistics Service of the Russian Federation). SPARK-Interfax online media was a source of the initial information on registration data of companies and their financial statements [5].

The sample was made without taking into account the attribute of the “operating” company. As of the day when the data were summarized and annual consolidated registers were made, repetitions were impossible. The scope of the empirical survey made it possible to include objects of various organizational and legal forms, market and service-oriented: a) legal entities that are profit-making corporations (in a wide sense); b) legal entities that are non-profit-making corporations (in a narrow sense); c) unincorporated organizations (in a narrow sense); d) international organizations that operate in Russia (in a narrow sense); e) legal entities that are commercial unitary organizations (in a narrow sense); f) legal entities that are non-commercial unitary organizations (in a narrow sense).

The sample did not include organizational and legal forms of individuals (entrepreneurs) except for a few earlier evaluation periods, but truncated. To achieve the consistency of the database, the regional structure of objects was captured in the survey in the format available prior to changes that had entered into force in 2014.

The data have covered almost all the types of market-oriented economic activities according to the classifier, excluding “Activities of extraterritorial organizations and bodies”, as well as “Undifferentiated activities of particular households that produce goods and services for own consumption”.

### 3.2 Sample

To assess the sample quality, the limiting error was calculated for a proportion without repeated observations for 2002–2015 with various accuracy of figures (90–99%) (Table 10). In all of the cases, the actual error was not over 3% with the average value of Δ*p* in 2.24% accurate within 99%. The total sample size per year was 54,229–126,953 companies. In total, in 1991–2015, the sample size was 24,236–126,953 with 75,958 as an average number of companies per sample per year. The low limiting error indicates that the sample and the performed empirical survey are representative.

**Table 10:** Quality of sample for 2002–2015.

Year	Sample size (companies)	Feature share	Actual limiting error (Δ*p*),%		
			*P*=90%	*P*=95%	*P*=99%
2002	82,391	0.5	1.065	1.269	1.668
2003	78,074	0.5	1.757	2.094	2.752
2004	76,621	0.5	1.780	2.121	2.787
2005	91,921	0.5	1.791	2.134	2.804
2006	126,953	0.5	1.575	1.877	2.467
2007	102,274	0.5	1.689	2.012	2.644
2008	97,708	0.5	1.758	2.094	2.752
2009	83,703	0.5	1.536	1.831	2.406
2010	93,314	0.5	1.441	1.718	2.257
2011	98,786	0.5	1.088	1.296	1.704
2012	94,784	0.5	0.869	1.036	1.362
2013	88,881	0.5	1.071	1.276	1.678
2014	81,679	0.5	1.027	1.224	1.609
2015	54,229	0.5	1.616	1.925	2.530
*Mean limit error (Δp)*			*1.43*	*1.71*	*2.24*

*Note:P* – consistency/accuracy, Δ*p* – limiting error.

The data for each year were treated as an independent set of information (without repetitions) for a top-down assessment of the corporate survival rate. The horizontal analysis between years does not eliminate repeated data, so all the information is kept, providing the continuity in the review.

### 3.3 Empirical models

The author's approach, which allows adjusting the company's life length to limits of a *conditionally active time period*, during which organizations show signs of doing business, is a distinctive feature of an empirical survey. It is worth mentioning that the cases are not rare when companies nominally exist without any transactions and this distorts a true picture using which it is possible to assess the overall survival rate of companies and their life length. The essence of the approach lies in a cross-join of two periods: 1) period of the legal life of the company from its incorporation to liquidation time; 2) period of business activities, which, within the framework of the survey, is assessed on the basis of financial statements provided by companies. To find the length, the priority is to have a year of incorporation as a left (start) point and a year of the company's last financial report as a right (end) point within the range. Such a combination needs to be checked for possible clerical mistakes (for instance, when a beginning year is later than an end-year - such mistakes were found for the companies, incorporation years of which were incorrectly specified on the information system). The 1987/1991–1998 sample resulted from the assumption because companies’ financial statements had been only available since 1999.

The assumption implies that the companies established in 1987–1998 continued their activities if there was an evidence that in 1999 and later they had transactions. This assumption also creates a blank space in an assessment of the corporate survival rate before 1999 but does not hamper a comprehensive analysis for the period of T+1 and longer with a time lag. The retrospective combination makes it possible to expand significantly the survey horizon up to 17 years.

### 3.4 Implications of study

The data obtained compensate for the paucity of the official statistics for the corporate survival rate in Russia. They are an important tool for implementing cross-country comparisons and analyzing the “the strength” of the economic system. The data serve as a benchmark to measure a degree of the market development and competitiveness among companies. The assessment of the current business conditions in the corporate survival rate exhibits the policy favouring business initiatives and entrepreneurship. At the same time, analysing dynamics can be of use in indicating the periods of economic downturn and stagnation, structural changes, regional differentiation, as well as complexity and uncertainty of the environment. Measuring the resistance rate of companies’ dynamic capabilities and duration of the memory effect of organizational behaviour under external negative and positive conditions is one of the promising areas for application of these data. The research expands the dataset for economic statistics and analysis, as well as implements the approach of alternative verification and refinement of the findings of other studies in the field of survival rate and lifecycle of companies in Russia.
